# Integrated Model for Evidence-Based Risk Factor Prioritisation and Dynamic Resource Allocation in Hypertension Prevention and Control: A Study Protocol

**DOI:** 10.3390/healthcare14080988

**Published:** 2026-04-09

**Authors:** Martins Nweke, Julian Pillay

**Affiliations:** Department of Basic Medical Sciences, Durban University of Technology, Durban 4001, South Africa; pillayjd@dut.ac.za

**Keywords:** hypertension, risk factors, resource allocation, DALYs, evidence-based policy, optimisation, South Africa

## Abstract

**Highlights:**

**What are the main findings?**

- This study introduces a Risk Priority Score that merges three independent dimensions, namely causality strength, implementation readiness, and contextual feasibility rather than relying only on burden or cost-effectiveness estimates as seen in GBD and WHO-CHOICE.
- The Causality Index advances current approaches by weighting the effect size, certainty of evidence, study design, and heterogeneity, creating a structured measure that resolves the limitations of single-point relative risks used in existing models.
- The Translation Readiness Index brings implementation maturity into the prioritisation process, which is absent in GBD and not formally incorporated in WHO-CHOICE modelling.
- The Implementation Priority Scale adds four nationally relevant dimensions—feasibility, scalability, policy integration, and equity—providing a context-specific layer that is not captured in global models.

**What are the implications of the main findings?**

- The composite Risk Priority Score offers a transparent and reproducible mechanism for ranking risk factors, improving on prior systems that rely on burden-only or cost-only metrics.
- The resource allocation model introduces an equity-weighted optimisation structure that adjusts DALY gains according to underserved population groups, addressing a major gap in standard WHO-CHOICE cost-effectiveness tools which treat all beneficiaries identically.
- The integration of prioritisation and optimisation into a single workflow creates a decision-support mechanism that allows policymakers to test scenarios and see how priorities shift under different budget and equity conditions.
- The model is designed for national and provincial application, making it the first South African hypertension model that connects local epidemiological evidence with dynamic resource allocation decisions.

**Abstract:**

**Background:** Hypertension remains one of the leading causes of cardiovascular morbidity and mortality in South Africa. Although extensive evidence exists on modifiable risk factors, the translation of this evidence into strategic and equitable health investments remains limited. Current models such as the Global Burden of Disease (GBD) and WHO “Best Buys” identify key exposures, but lack operational mechanisms for context-specific prioritisation and dynamic resource allocation. The aim of this study is to develop and validate an integrated decision-support model that links evidence-based risk factor prioritisation with dynamic budget allocation to improve hypertension prevention and control in South Africa. **Methods:** This study adopts a two-phase mixed-methods design. Phase 1 develops a Risk Factor Prioritisation Model that ranks modifiable exposures using composite indices for the causality strength, implementation feasibility, policy integration, and equity. Phase 2 constructs a Dynamic Resource Allocation Model that distributes health budgets across interventions to maximise Disability-Adjusted Life Years (DALYs) averted, subject to budget and equity constraints. The model integrates data from systematic reviews, GBD 2019 estimates, WHO-CHOICE cost data, and national health expenditure databases. A validated quantitative Risk Priority Score (RPS) for major hypertension risk factors, an optimisation model for resource allocation, and an interactive dashboard that visualises efficiency and equity trade-offs under varying budget scenarios are expected. **Conclusions:** This study will provide a reproducible model for transforming epidemiological and economic evidence into actionable policy guidance. It bridges the gap between evidence generation and health planning, supporting more equitable and data-driven decision making in noncommunicable disease control.

## 1. Introduction

Hypertension is among the most prevalent and preventable causes of morbidity and mortality worldwide [1]. Clinically, it is defined as a sustained elevation of arterial blood pressure resulting from complex interactions between genetic predisposition, neurohormonal dysregulation, vascular dysfunction, renal sodium handling, and environmental exposures [2]. The majority of cases are classified as primary (essential) hypertension, while secondary hypertension arises from identifiable causes such as chronic kidney disease, endocrine disorders, or vascular abnormalities [3]. Regardless of the aetiology, persistently elevated blood pressure accelerates vascular injury, promotes left ventricular hypertrophy, and induces progressive damage to target organs [4,5].

Uncontrolled hypertension is a major upstream driver of cardiovascular disease, stroke, heart failure, and chronic kidney disease, and it contributes substantially to cognitive decline and premature mortality [6]. These pathological consequences develop insidiously, often in asymptomatic individuals, leading to delayed diagnosis and prolonged exposure to risk [2]. The effective control of blood pressure through lifestyle modification and pharmacological therapy has been shown to markedly reduce the incidence of stroke, myocardial infarction, and renal failure, underscoring hypertension as a highly tractable target for prevention [7,8].

Globally, more than 1.3 billion adults are living with hypertension, with over two-thirds residing in low- and middle-income countries (LMICs) [9,10]. Sub-Saharan Africa bears a disproportionate share of this burden, with some of the highest prevalence rates worldwide and among the lowest levels of awareness, treatment, and control. Fewer than one in three individuals with hypertension in the region achieve adequate blood pressure control, reflecting gaps in screening, continuity of care, and long-term adherence [1,11,12]. In South Africa, hypertension is a leading contributor to premature mortality and disability, accounting for approximately 13% of all deaths and more than two million disability-adjusted life years (DALYs) annually [13,14].

South Africa’s hypertension profile has several distinctive clinical and epidemiological features [15]. Hypertension tends to present earlier in adulthood, is more severe, and is less well controlled among Black African populations compared with White populations, a pattern shaped by both biological susceptibility and structural disadvantage [16]. Higher salt sensitivity, a greater prevalence of obesity in women, lower rates of consistent treatment, and barriers to sustained care contribute to these disparities [17]. In contrast, White South Africans exhibit lower prevalence and higher control rates, reflecting differential access to healthcare, socioeconomic conditions, and health-seeking behaviour [18,19,20]. These inequalities are further reinforced by rapid urbanisation, food-system transitions, harmful alcohol use, physical inactivity, and occupational exposures such as mining-related dust, all of which interact with poverty and limited educational attainment [21,22,23].

Despite the availability of effective antihypertensive medications and evidence-based behavioural interventions, progress in population-level control has remained slow [24,25]. Health system constraints, including inconsistent screening, fragmented primary care, medication stock-outs, and poor long-term adherence, continue to undermine the treatment effectiveness [26,27]. At the policy level, these challenges are compounded by inefficiencies in health budget allocation and the absence of transparent, evidence-based mechanisms to guide investment toward interventions that are not only effective, but feasible, scalable, and equitable [1,8].

Over the past two decades, extensive observational studies and meta-analyses have quantified the contribution of modifiable risk factors to hypertension, including a high sodium intake, excess body weight, physical inactivity, alcohol consumption, and tobacco use [19,28,29]. While these studies clarify causal pathways, they are rarely translated into structured frameworks for prioritising interventions or guiding the allocation of limited resources. Most systematic reviews conclude with pooled relative risks or odds ratios, offering little practical guidance on how such evidence should shape policy or budgetary decisions in LMIC settings. This limitation sustains a persistent gap between scientific evidence and effective public health action [30,31].

The Global Burden of Disease (GBD) framework provides the most comprehensive quantification of the hypertension burden and its attribution to modifiable risk factors using a comparative risk assessment expressed in DALYs [32,33]. However, the GBD is primarily descriptive and not designed to support national-level prioritisation or optimise investment decisions within constrained health budgets [34,35]. Similarly, the World Health Organisation’s “Best Buys” for noncommunicable disease prevention identify cost-effective interventions but do not offer an adaptive mechanism for balancing multiple interventions, addressing provincial variation, or accounting for entrenched social inequities [1,36].

To address these limitations, this study introduces an integrated model for evidence-based risk factor prioritisation and dynamic resource allocation in hypertension prevention and control. The model extends comparative risk logic by incorporating causal strength, implementation readiness, and equity considerations into a unified prioritisation framework. These priority scores are then linked to a dynamic allocation engine that applies optimisation modelling to distribute finite resources in ways that maximise DALYs averted while promoting fairness across provinces and population groups.

By integrating meta-analytic evidence, national implementation indicators, and cost-effectiveness modelling, the proposed framework moves beyond a descriptive risk assessment toward actionable decision support. It aims to generate a validated, context-specific model that enables policymakers to simulate allocation scenarios, assess trade-offs between efficiency and equity, and identify strategies most likely to reduce South Africa’s hypertension burden. Ultimately, this study seeks to support optimal and equitable investment in hypertension prevention and control, with relevance for other noncommunicable diseases and comparable LMIC contexts.

## 2. Materials and Methods

### 2.1. Study Design

This study adopts a two-phase study, namely systematic review with meta-analysis (SLR) and record review of quantitative evidence on cost, coverage, and policy implementation of hypertension control in South Africa. The systematic evidence synthesis will be linked to a record review-driven quantitative optimisation model to guide hypertension-related investment decisions in South Africa.

- Phase 1 develops and validates a Risk Factor Prioritisation Model (RPF) for hypertension based on meta-analytic and contextual indicators.
- Phase 2 constructs and tests a Dynamic Resource Allocation Model (DRAM) that converts these priority scores into optimal funding distributions across interventions to maximise Disability-Adjusted Life Years (DALYs) averted while promoting equity.

The approach follows the comparative risk logic of the Global Burden of Disease (GBD) model [28,29] and the cost-effectiveness conventions of WHO-CHOICE [21].

### 2.2. Study Setting

The analytical model is tailored to the South African health system context, focusing on national and provincial levels. Hypertension prevention and control programmes within the public sector will serve as the reference environment for estimating feasibility, cost, and policy integration parameters. Data will be drawn from national surveys and administrative reports representing all nine provinces and diverse settlement types (urban, peri-urban, and rural). This study will be carried out in the Globa Health Unit, Department of Basic Medical Science, Faculty of Health Sciences, Durban University of Technology.

Population/unit of analysis

In this study, the unit of analysis comprises the peer-review literature that examined the associations between hypertension and biopsychosocial determinants in South Africa, and records on cost, coverage, and policy implementation of hypertension control and prevention in South Africa. For the SLR, observational quantitative observational studies will be included, irrespective of the study design. This is because case-control and cohort studies are very few in South Africa. However, differences in design will be accounted for when computing causality index. For, the record review, only data reported in accredited national bodies such as Health National Department of Health, Stats SA, Health Systems Trust, WHO, and Global Burden of Disease will be included. Recent reports will be preferred to older ones.

### 2.3. Data Items and Sources

In Phase 1, we will integrate multiple categories of data drawn from the peer-reviewed literature, national surveys, and global policy databases to construct the Risk Factor Prioritisation Model (RPF). Each data stream contributes to one or more indices—the Causality Index (CI), Translation Readiness Index (TRI), and Implementation Priority Scale (IPS)—which collectively generate the Risk Priority Score (RPS) for each modifiable risk factor.

Effect estimates, expressed as pooled relative risks (RR) or odds ratios (OR), will be extracted from published primary studies exploring the predictors of hypertension in South Africa between 2000 and 2025 indexed in PubMed, Scopus, Web of Science, and African Journals Online. These estimates will quantify the causal strength of association between exposures, such as high sodium intake, obesity, harmful alcohol use, tobacco consumption, physical inactivity, and low fruit and vegetable intake, and hypertension incidence or mean blood pressure change [5,15,21,30]. The pooled estimates and heterogeneity statistics (I^2^) from these studies will inform the computation of the Causality Index (CI), weighted by design and quality of evidence [33].

National exposure distributions and Disability-Adjusted Life Years (DALYs) attributable to each risk factor will be derived from the Global Burden of Disease 2019 dataset for South Africa [1]. These data will be used both to validate the RPS hierarchy and to assess alignment with comparative risk rankings in the broader GBD model [27,28]. Contextual indicators describing feasibility and scalability will be obtained from the South African Demographic and Health Survey 2016 [34], the South African National Health and Nutrition Examination Survey (SANHANES-1) 2013 [35], and cost-coverage reports of the HST 2022 [36]. These national datasets capture service availability, programme reach, and operational constraints across provinces and facility levels [8,9].

Evidence of policy integration will be drawn from the National Strategic Plan for the Prevention and Control of Noncommunicable Diseases 2022–2027 [37] and the National Hypertension Guidelines 2023 [38] to determine the extent to which interventions targeting each risk factor have been institutionalised in existing public health models [14,38]. Indicators of equity including differences in exposure, treatment access, and outcomes across sex, income, and geography will be calculated from the Statistics of South Africa General Household Survey 2022 [35,39] and subnational GBD 2019 estimates [15]. These data will quantify equity gradients and inform the weighting of the Equity component within the IPS [19].

Assessment of translation readiness will use implementation maturity classifications from the WHO “Best Buys” and Other Recommended Interventions’ 2014 and 2023 updates [20], and reports of the National NCD Programme [37]. Each intervention will be scored as 0 (experimental), 1 (pilot/limited implementation), or 2 (nationally implemented) to compute the Translation Readiness Index (TRI) [20]. Economic and programme coverage data supporting feasibility and later linkage with the allocation model will come from the Health Systems Trust cost databases and WHO-CHOICE regional cost-effectiveness analyses [40]. Quality assessment variables, including risk of bias score and heterogeneity indices, will provide study-weighting parameters for each pooled estimate.

These datasets will, collectively, supply the empirical foundation for calculating the Risk Priority Score (RPS)—a composite measure integrating causal strength, implementation maturity, and contextual feasibility for each modifiable hypertension risk factor in South Africa (Table 1).

### 2.4. Analytical Procedure

The analytical process will combine quantitative meta-analytic evidence with contextual and policy indicators to derive a unified Risk Priority Score (RPS) for each modifiable hypertension risk factor in South Africa. The procedure involves four main stages: computation of the Causality Index (CI), Translation Readiness Index (TRI), Implementation Priority Scale (IPS), and final RPS aggregation and validation.

Computation of the Causality Index (CI)

For each exposure–hypertension relationship, pooled effect estimates will be extracted from eligible studies [33]. Relative risks (RRs) or odds ratios (ORs) will be standardised on a natural-log scale and the corresponding 95% confidence intervals will be used to assess statistical precision. When multiple meta-analyses exist for a given exposure, the most recent and methodologically robust estimate—based on GRADE quality ratings—will be retained. We will account for study design/temporality and heterogeneity using weighted scoring system. We defined temporality as the ratio of RCT and cohort studies to total studies included in the meta-synthesis of a given factor. Temporality was excellent if the ratio was ≥0.75 (value 3), fair if the ratio ranged between 0.74 and 0.5 (value 2), and poor if the ratio was <0.5.

The Causality Index (CI) quantifies the causal attribute of a risk factor (r) by combining effect magnitude, risk responsiveness, temporality, and certainty of the evidence underlying a risk [33]. It is computed using a 10-point scale as follows:

$$CI\_r=10\times s\_m\times (1-I^{2}\_r/100)\times w\_c\times w\_q$$

where:

- s_m = scaled magnitude term, calculated as s_m=min(1,ln(OR_r)/ln(OR_anchor)), with OR_anchor set to 3.0;
- I^2^_r = heterogeneity statistic (0–100);
- w_c = certainty weight (1.0 for high, 0.75 for moderate, 0.5 for low, and 0.25 for very low certainty);
- w_q = design/temporality weight (1.0 for cohort/RCT, 0.75 for case–control and 0.5 for cross-sectional).

Importantly, this formulation penalises heterogeneous or low-certainty evidence while ensuring comparability across exposures. Hence, risk factors will then be classified as:

- First-class (CI ≥ 7): strong, consistent evidence of causality;
- Second-class (CI = 5–6): moderate evidence;
- Third-class (CI ≤ 4): weak or inconsistent evidence.

Computation of the Translation Readiness Index (TRI)

The TRI measures the extent to which effective interventions addressing each risk factor have been developed, tested, and scaled. Evidence will be drawn from WHO “Best Buys,” [20] national NCD strategies, and programme reports [37]. Each risk factor will be scored from 0 to 2:

- 0 = experimental/limited evidence;
- 1 = pilot or local implementation;
- 2 = implemented nationally or included in policy.

Computation of the Implementation Priority Scale (IPS)

The IPS captures contextual feasibility and policy relevance through four domains: Feasibility, Scalability, Policy Integration, and Equity. Each domain is rated from 0 to 2 (low priority to high priority) based on national and provincial evidence:

- Feasibility: availability of infrastructure, human resources, and supply chains;
- Scalability: ability to expand intervention coverage at reasonable cost;
- Policy Integration: alignment with national strategies and legislative support;
- Equity: capacity to reduce exposure or disease burden among vulnerable groups.

The IPS scores will be normalised to a 0–2 range using the formula:

IPS_r=[(F_r+S_r+P_r+E_r)/8]×2

where F_r, S_r, P_r, and E_r denote scores for each domain.

Computation of the Composite Risk Priority Score (RPS)

The RPS integrates the three indices (CI, TRI, and IPS) into a single numeric measure reflecting both scientific evidence and practical implementability.

RPS_r=(CI_r+TRI_r+IPS_r)/3

Each RPS value will be rescaled to a 0–2 (low-risk to strong-risk priority) interval to ensure comparability across risk factors. A higher score indicates greater causal strength, readiness, and contextual relevance, signifying higher implementation priority.

Illustrative Example for CI and RPS Computation

To improve clarity, this subsection provides a simple example showing how the Causality Index (CI) and the final Risk Priority Score (RPS) are calculated.

**Step 1: Compute the Causality Index (CI)**

Assume a hypothetical risk factor with the following characteristics:

- Pooled OR = 2.0;
- Certainty of evidence = moderate;
- Study design mix includes cohort and case-control studies;
- Heterogeneity (I^2^) = 40%.

Using the CI components:

1. **Magnitude term (s_m_)**

The effect size is scaled relative to the anchor value (OR = 3.0).

A pooled OR of 2.0, therefore, produces a magnitude score between 0.5 and 0.7 on the scale.

2. **Certainty weight (w_c)**

Moderate certainty assigns a weight of 0.75.

3. **Design/temporality weight (w_q)**

Mixed longitudinal and case-control designs assign a weight of 0.75.

4. **Heterogeneity penalty**

I^2^ of 40% produces a modest deduction.

When components are combined, the example risk factor obtains a CI of **6.2**, placing it in the “moderate evidence” category.

**Step 2: Compute the Translation Readiness Index (TRI)**

Suppose the intervention addressing this risk factor is already piloted in several provinces but not yet nationally scaled. This corresponds to a TRI score of **1**.

**Step 3: Compute the Implementation Priority Scale (IPS)**

Assume the four IPS domains score as follows:

- Feasibility = 2;
- Scalability = 1;
- Policy integration = 1;
- Equity contribution = 2.

The average score is then normalised into the 0–2 range, giving an IPS of **1.5**.

**Step 4: Combine CI, TRI, and IPS into the RPS**

The RPS aggregates all three components and rescales to a 0–2 scale.

For this example, the combined value produces a normalised RPS of approximately **1.4**, indicating high priority for implementation under South African conditions.

Validation and Sensitivity Analyses

The RPS ranking for the identified factors will be compared with the GBD South Africa population attributable fraction hierarchy for the same factors using Spearman’s ρ to assess concordance. Also, the results will be benchmarked against WHO-AFRO and regional burden-of-disease estimates to test generalisability [28,42]. Sensitivity testing: scenario analyses will re-weight individual components (for example, doubling the weight of the Equity or Feasibility domain) to evaluate stability of rankings and identify factors most influencing prioritisation outcomes.

Handling of Missing and Outdated Data

The model relies on multiple secondary datasets, and several steps will be taken to manage gaps, inconsistencies, or older estimates, especially at provincial level. When provincial values are not available for a given indicator, the model will draw on the nearest reliable national estimate and adjust it using proportional differences observed in related variables such as prevalence, service coverage, or demographic composition. Where two or more provincial datasets report conflicting values, the more recent and methodologically transparent source will be used. For variables with incomplete temporal coverage, trends from GBD and national surveys will guide simple interpolation, ensuring that values remain within plausible bounds. Sensitivity analyses will test how alternative assumptions influence the Risk Priority Score and allocation outcomes. This approach maintains the internal coherence of the model while acknowledging the practical limitations of existing epidemiological and administrative data.

**Phase 2: Dynamic Resource Allocation Model (DRAM)**

Phase 2 translates the prioritised risk factors identified in Phase 1 into an optimisation model that recommends how limited budgets for hypertension control should be distributed across interventions. The model aims to maximise Disability-Adjusted Life Years (DALYs) averted within fixed resource constraints while ensuring fairness in allocation between provinces and population groups.

**Model Structure**

The Dynamic Resource Allocation Model (DRAM) applies a linear programming formulation consistent with cost-effectiveness and resource allocation methods described in the WHO-CHOICE model and related health–economic studies [20,30,45].

Maximise Z=Σ(RPS_r×DALY_r×x_r)

Subject to the constraints:

Σ(Cost_r×x_r)≤B,x_r≥0

where:

RPS_r: Risk Priority Score for factor r (from Phase 1);
DALY_r: DALYs averted per coverage unit;
Cost_r: unit intervention cost;
x_r: allocation share;
B: total hypertension budget.

To capture distributional fairness, an equity-weighting parameter (μ) modifies each intervention’s effectiveness:

DALYadj,r=DALY_r(1+μ_r)

This adjustment, adapted from equity-weighted cost-effectiveness models [32,46], gives higher value to interventions benefitting underserved populations.

### 2.5. Parameter Estimation

Cost inputs: Unit costs are drawn from the WHO-CHOICE African Region database [13], the Health Systems Trust District Health Barometer 2022) [36], and the National Department of Health (NDoH), 2023 financial reports [41]. All values are expressed in 2024 Rand (ZAR) using the CPI Health sub-index [39].

Effectiveness inputs: DALYs averted per intervention are derived from GBD 2019 comparative risk data [1,33] and national studies of hypertension intervention impact [9,20]. When local DALY values are unavailable, relative risk reductions from the Causality Index are scaled to the DALY burden for that risk factor [27].

Equity parameter (μ): μ-weights are estimated from provincial income quintiles and rural–urban coverage gaps reported in the South African Demographic and Health Survey 2016 [34] and SANHANES-1 2013 [35]. Following Hauck et al. [47], μ ranges from 0 for fully served populations to 0.4 for underserved rural or low-income communities.

Budget (B): Three budget bands are modelled: low (R 50 million), medium (R 100 million), and high (R 200 million). These reflect realistic national and provincial scenarios within South Africa’s NCD funding structure [37,48].

Implementation and Software

The optimisation will be implemented in R (version 4.4) using lpSolve, ROI, and ggplot2 packages. Results will be displayed in an interactive dashboard (Streamlit interface or Excel template), illustrating:

- Optimal allocation percentages by risk factor;
- DALYs averted under efficiency-only and equity-weighted conditions;
- Marginal returns and frontier curves;
- Scenario comparisons across budget bands.

This approach follows principles for transparent and reproducible economic modelling [49,50].

Validation and Sensitivity Testing

Internal validation: compare model allocations with WHO “Best Buy” benchmarks for hypertension interventions [43,44]. Scenario testing: ±20% variation in cost and DALY inputs to evaluate stability. Equity sensitivity: increase μ weights for rural provinces to observe redistributive effects on total DALYs. Cross-validation: repeat optimisation using alternate RPS normalisations (0–1, 0–5, and 0–10) to test structural robustness [40].

External Validation and Stakeholder Input

In addition to internal validation against WHO “Best Buys” and GBD comparative-risk rankings, the model will undergo external review by experts familiar with hypertension prevention and health-planning processes in South Africa. This will include consultation with technical staff within the National Department of Health, as well as academic and provincial stakeholders involved in NCD programming. These consultations will focus on the interpretation of the Risk Priority Score, the practical relevance of the feasibility and policy indicators, and the usability of the allocation dashboard. Feedback will be used to refine assumptions, adjust parameter ranges where necessary, and ensure that the model aligns with real-world decision structures. This external input strengthens the validity of the model and supports its eventual uptake within routine planning cycles.

### 2.6. Ethical Considerations

As this study uses publicly available and administrative datasets (GBD, WHO-CHOICE, SADHS, SANHANES, and HST), no individual-level identifiers will be accessed. Institutional ethics approval has already been secured from the Durban University of Technology Research Ethics Committee as this study is part of larger hypertension study in South Africa (Ethical Clearance number: IN-IREC 025/25). Data handling will comply with the Protection of Personal Information Act (POPIA), 2013 and international research data management standards.

## 3. Results

Phase 1 will generate a validated, evidence-based hierarchy of modifiable hypertension risk factors in South Africa. Using pooled effect estimates from a meta-analysis [50] and national datasets such as SANHANES-1 2013 and the South African Demographic and Health Survey 2016, this study will compute three composite indices—the Causality Index (CI), Translation Readiness Index (TRI), and Implementation Priority Scale (IPS)—which will be combined to form a composite Risk Priority Score (RPS) scaled between 0 and 2. The RPS table will rank exposures such as a high sodium intake, obesity, harmful alcohol use, tobacco consumption, physical inactivity, and low fruit and vegetable intake according to both causal strength and implementation feasibility [9,22,51]. Validation will be undertaken through comparison with the GBD 2019. The South Africa hierarchy will be assessed using Spearman’s ρ, while sensitivity analyses will assess the robustness of the weighting and normalisation schemes [24]. Additional outputs from Phase 1 will include an interactive evidence matrix summarising the RPS values and policy alignment, a structured dataset containing pooled effect sizes, a certainty of evidence and temporality index (I^2^), and a concise policy brief identifying high-, moderate-, and low-priority risk factors for integration into national hypertension control strategies [24,37]. These outcomes will inform the empirical foundations of Phase 2.

Phase 2 will operationalise the findings of Phase 1 through the Dynamic Resource Allocation Model (DRAM)—a linear-programming optimisation model designed to allocate hypertension-control budgets efficiently and equitably. The DRAM will use the unit cost and DALY data from WHO-CHOICE [20,34], Health Systems Trust 2022, and national cost-effectiveness studies [9,20], integrating an equity parameter (μ) that prioritises underserved populations [40,47,49]. Expected outputs include an optimised allocation matrix indicating funding shares per risk factor, equity-adjusted efficiency frontiers illustrating trade-offs between cost-effectiveness and fairness, and a scenario dashboard for dynamic policy testing. The final deliverable will be a policy brief summarising DALYs averted, equity impacts, and implications for resource distribution, aligned with South Africa’s National Strategic Plan for NCDs (2022–2027) [37,49].

**Figure 1:**
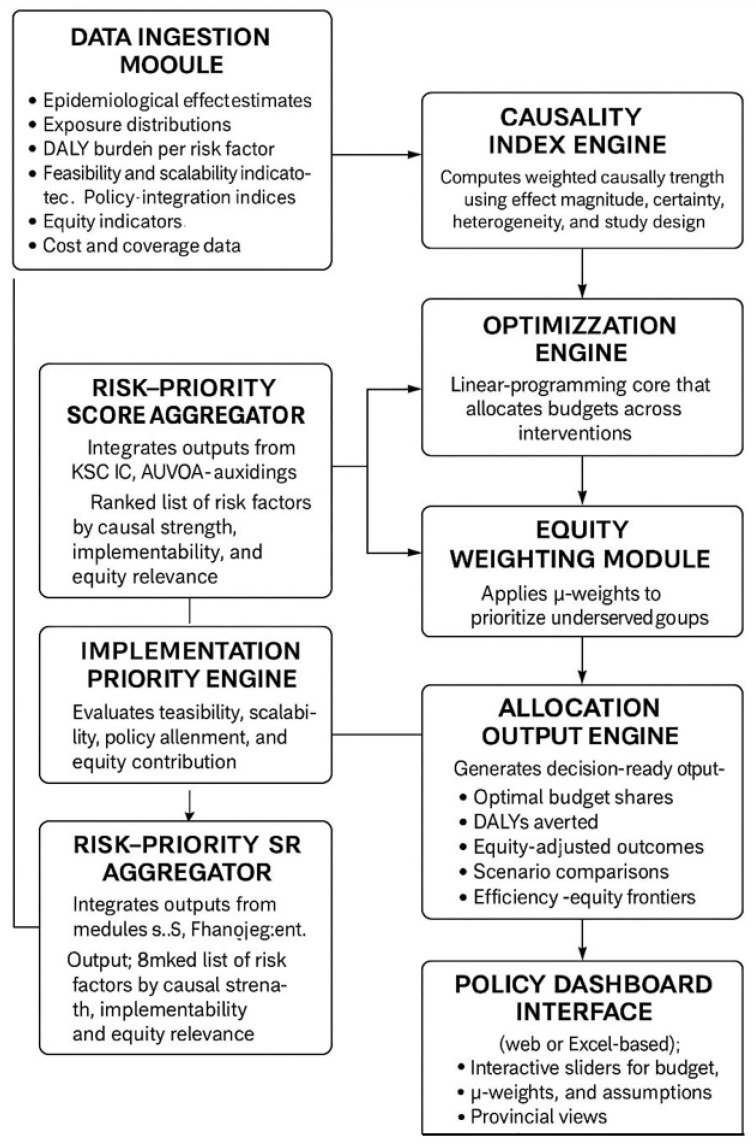
Integrated decision support model.

The entire study is expected to be completed within twelve months. The literature synthesis, data extraction, and computation of the indices will occupy the first four months, followed by analysis and validation in month five. The DRAM implementation, parameter estimation, and optimisation modelling will occur between months six and nine, and policy translation, manuscript drafting, and dissemination will conclude by month twelve. The combined phases will deliver an integrated decision-support model (Figure 1) linking meta-analytic evidence with dynamic resource allocation modelling to enhance hypertension prevention and control in South Africa [19,38,49].

## 4. Discussion

Hypertension remains one of the leading contributors to cardiovascular disease and premature mortality in low- and middle-income countries (LMICs), where health systems face persistent resource and capacity constraints [5,9]. Despite the availability of effective interventions, including sodium reduction, antihypertensive therapy, and lifestyle modification, coverage and control rates remain suboptimal across sub-Saharan Africa [10,14]. In South Africa, where over one in three adults is hypertensive, the challenge lies not in the absence of evidence, but in the absence of mechanisms that translate evidence into fair and efficient investment decisions [12,37].

This study proposes an integrated, evidence-based model for strengthening hypertension prevention and control in South Africa through a two-phase design that links systematic risk factor prioritisation with dynamic resource allocation. Unlike conventional descriptive burden assessments, the model combines causal epidemiological evidence with implementation readiness and equity metrics, providing a structured pathway from research synthesis to actionable policy modelling.

The proposed Risk Factor Prioritisation Model (RPF) addresses this gap by ranking risk factors according to both their causal strength and practical feasibility for intervention. The inclusion of indices such as the Causality Index (CI) and Implementation Priority Scale (IPS) represents a methodological advance over traditional burden metrics, which often neglect context-specific implementability. By integrating evidence from meta-analyses, national datasets, and policy documents [21,22,34], the model ensures that prioritisation is not only scientifically grounded, but also responsive to South Africa’s policy environment.

The second phase—namely the Dynamic Resource Allocation Model (DRAM)—builds on this foundation by introducing optimisation and equity weighting into resource allocation decisions. Linear programming models have been widely used in global health economics to maximise health outcomes under budget constraints [40,49]. However, their application to hypertension control at a national level in LMICs remains limited. By incorporating an equity parameter (μ) that adjusts for underserved populations, the DRAM aligns with calls for fairness in universal health coverage planning [47,48]. This structure allows policymakers to visualise trade-offs between cost-effectiveness and equity, an essential consideration in contexts marked by socioeconomic and geographic disparities.

Collectively, these two phases represent an operationalisation of the knowledge-to-action model advocated in implementation science [25,26]. The approach enables a direct link between epidemiological synthesis, policy feasibility assessment, and data-driven resource optimisation. The expected outputs—validated risk factor rankings, an optimised allocation matrix, and interactive dashboards—will facilitate an evidence-informed dialogue among health planners, economists, and policymakers. The dashboard is intended for public release as part of the project’s dissemination package. Policymakers will be able to access visual summaries of priority rankings, allocation options, and equity impacts. Provinces will also be able to adapt the model to their own data. In this way, the model becomes a practical and reusable decision-support tool that aligns closely with existing planning cycles and enhances transparency in resource allocation.

Some limitations are anticipated. First, the analysis will depend on secondary data sources, which may vary in quality and temporal alignment. Second, meta-analytic estimates may not capture provincial or demographic heterogeneity in risk exposure. Third, the optimisation model assumes linear relationships between investment and DALYs averted, which may simplify real-world interactions among interventions. To mitigate these limitations, sensitivity analyses and alternative weighting schemes will be employed.

Notwithstanding this, the integrated model to be proposed has broader implications beyond hypertension. Its adaptable design means it can be extended to other non-communicable diseases, including diabetes, dyslipidaemia, and stroke, using similar logic for causal weighting and equity adjustment. By embedding reproducibility through open-source R (version R 4.3) and Python scripts (version Python 3.10), the project also contributes to transparency in health system modelling, aligning with global movements toward open science and policy accountability [43,44].

## 5. Conclusions

This study seeks to bridge the persistent divide between epidemiological evidence and actionable policy for hypertension control. By integrating systematic review methods with dynamic modelling, it will provide South Africa with a transparent, equitable, and data-driven model for prioritising interventions within constrained budgets. If validated, the model could serve as a scalable blueprint for national and regional decision-support systems targeting non-communicable diseases in similar resource-limited settings.

## Figures and Tables

**Table 1:** Data items/variables and sources.

Data Item	Purpose/Variable Captured	Source	Type of Data
1. Effect estimates (RR/OR)	Quantify strength of association between each modifiable exposure and hypertension (for Causality Index—CI)	Peer-reviewed epidemiological studies indexed in PubMed, Scopus, Web of Science, Academic Search Complete, and African Journals Online	Secondary quantitative
2. Exposure distribution by risk factor	National prevalence of obesity, high sodium intake, alcohol use, tobacco use, physical inactivity, and low fruit and vegetable intake	GBD 2019 country-level exposure dataset for South Africa [1]	Secondary quantitative
3. DALYs attributable to each risk factor	Measure of health loss (used for validation against RPS order)	GBD 2019 Risk Factors Collaborators dataset and GBD Results Tool [1]	Secondary quantitative
4. Feasibility indicators	Availability of interventions, delivery infrastructure, and implementation challenges	South African Demographic and Health Survey 2016 [34]; SANHANES-1 2013 [35]; District Health Barometer 2022 [36]	Survey/programme data
5. Scalability indicators	Geographic coverage and cost efficiency of intervention delivery	Health Systems Trust reports [36]; National Department of Health Annual Performance Plans 2018–2024 [41]	Administrative reports
6. Policy integration evidence	Presence of the risk factor or intervention in national policies or guidelines	National Strategic Plan for NCDs 2022–2027 [37]; National Hypertension Guidelines 2023 update [38]	Policy documents
7. Equity parameters	Disparities in exposure and intervention coverage by province, sex, and income quintile	Stats SA General Household Survey 2022 [39]; SANHANES-1 2013 [35]; GBD 2019 subnational estimates [1]	Survey/population
8. Implementation maturity (TRI)	Stage of intervention development or integration into national programming	WHO “Best Buys” and Recommended Interventions 2014 edition and 2023 update [41,42,43,44]; National NCD Programme reports [37]	Global and national policy data
9. Cost and coverage data	Inputs for feasibility and for later linkage with allocation model	Health Systems Trust cost databases; WHO-CHOICE regional cost effectiveness data	Economic/programme
10. Quality and heterogeneity metrics	Weighting of meta-analytic evidence in CI calculation	GRADE; I^2^ statistics from meta-analyses	Secondary review data

## Data Availability

No new data were created or analyzed in this study. Data sharing is not applicable to this article.
